# Should Thyroid Lobectomy Be Performed as a Day-Case Procedure? A Single-Centre Retrospective Audit

**DOI:** 10.7759/cureus.31435

**Published:** 2022-11-13

**Authors:** Ysabelle Embury-Young, Fatemah Keshtkar, Graham Porter

**Affiliations:** 1 ENT, University Hospitals Bristol and Weston NHS Foundation Trust, Bristol, GBR; 2 Radiology, University Hospitals Bristol and Weston NHS Foundation Trust, Bristol, GBR; 3 Head and Neck Surgery, University Hospitals Bristol and Weston NHS Foundation Trust, Bristol, GBR

**Keywords:** hemithyroidectomy, ear nose and throat, ent, clincal audit, post-thyroidectomy complications

## Abstract

Introduction

The British Association of Endocrine and Thyroid Surgeons (BAETS) published guidance in 2012 advising against day-case thyroid lobectomy due to the unpredictable risk of post-operative haematoma. Due to the increasing pressures the National Health Service (NHS) faces, a position statement was released by BAETS in 2020 highlighting their support of day-case thyroid lobectomy in specific low-risk cases with the introduction of appropriate local protocols.

Objective

We aimed to assess post-operative complications of thyroid lobectomy at University Hospitals Bristol and Weston NHS Trust (UHBW), a tertiary centre for Otolaryngology, to consider the feasibility and safety of day-case surgery in our unit.

Methods

We conducted a retrospective audit of 140 elective thyroid lobectomy cases identified in the period of January 2017 to December 2018 at UHBW. Inclusion criteria included no previous thyroid surgery or major head and neck surgery, elective thyroid lobectomy procedure only, and surgery performed at UHBW by otolaryngology or maxillofacial teams. Exclusion criteria included emergency hemithyroidectomy; hemithyroidectomy as part of another procedure; and completion thyroidectomy.

Results

A total of 140 elective thyroid lobectomy procedures were performed between January 2017 and December 2018. Of them, 125 cases met inclusion criteria, and 15 cases were excluded: two emergency hemithyroidectomy, three incorrect coding, four hemithyroidectomy as part of another head and neck procedure, and six completion thyroidectomy. Mean age was 51.9 years (range: 20-88 years), with 30 (24.0%) male cases and 95 (76.0%) female cases. Mean in-patient stay was 1.4 days (range: 0-19 days), and mean hours in hospital were 34 hours. Two (1.6%) cases were discharged on the same day of their operation. Post-operative complications included five (4%) cases of post-operative haematoma, four (3.2%) cases of unilateral vocal cord palsy, one (0.8%) case of pneumonia, and three (2.4%) cases of wound infections. From those cases that developed post-operative haematoma, 80% (n=4/5) developed less than 24 hours after the procedure. One (20%) case of post-operative haematoma developed on day 6 after their procedure and required readmission for re-operation.

Conclusion

The unpredictable nature of post-operative haematoma poses a significant risk to patients and medicolegal implications. Our findings suggest that there were no predictable risk factors amongst our patient cohort and therefore day-case thyroid lobectomy should be avoided. Further research is required to identify low-risk patients who could benefit from day-case surgery.

## Introduction

Thyroid lobectomy, also known as hemithyroidectomy, is an operative procedure to remove one lobe of the thyroid gland. This can be performed for several reasons, including thyroid cancer, thyrotoxicosis, thyroid goitre, and thyroid nodule. When considering thyroid lobectomy as day-case surgery, the principal consideration is risk to patient safety. The development of a post-operative haematoma is a rare but unpredictable complication of thyroid surgery and has an associated risk of life-threatening acute airway compression [1]. Other complications of thyroid surgery include, but are not limited to, post-operative wound infection, hypocalcaemia, and recurrent laryngeal nerve injury [2].

The Association of Anaesthetists and the British Association of Day Case Surgery guidelines clearly define day-case surgery as "the patient is admitted and discharged on the same day" [3]. In 1990, the Audit Commission [4] published a nationwide strategy to increase day-case surgery units across the National Health Service (NHS). This aimed to increase the capacity for day-case surgery and improve cost-savings through reduced inpatient admissions [5]. In 2012, the British Association of Endocrine and Thyroid Surgeons (BAETS) commissioned a review to establish the feasibility of day-case surgery for elective thyroidectomies [6]. However, this concluded that the potential risk to patient safety posed by post-operative bleeding and hypocalcaemia outweighed financial benefits of day-case surgery. Similar consensus has been reached in Europe and the United States of America with emphasis on the selection of specific patient cohorts where post-operative risks are reduced. For example, the European Association Francophone de Chirurgie Endocrinienne advised that only “highly selected patients” should be considered for outpatient thyroidectomy, and if any complications were to arise, “the surgeon stands first line in responsibility” [7]. Similarly, the American Association of Endocrine Surgeons suggests the consideration of a number of factors, such as surgeon experience and patient preference, prior to deciding on day-case versus inpatient admission [8]. More recently, in 2020, a position statement released by BAETS stated that day-case thyroid lobectomy could be considered in “low-risk” cases, but this is a decision that needs to be taken by individual centres [9].

This retrospective audit aimed to assess the incidence of post-operative haematoma development as a post-operative complication of thyroid lobectomy procedures. This was to assess the safety and feasibility of performing this operation as a day-case surgery given the potential for cost-savings at Bristol Royal Infirmary, the tertiary head and neck surgery centre within University Hospitals Bristol and Weston NHS Trust (UHBW).

## Materials and methods

Elective unilateral thyroid lobectomy cases performed between January 2017 and December 2018 at UHBW were identified through coding and retrospectively analysed. Inclusion criteria were no previous thyroid surgery or major head and neck surgery, elective thyroid lobectomy procedure only, and surgery performed at UHBW by otolaryngology or maxillofacial teams. Exclusion criteria included emergency hemithyroidectomy, hemithyroidectomy as part of another procedure, and completion thyroidectomy.

Pre-operative data analysed included age, sex, smoking status, reason for hemithyroidectomy, comorbidities, antiplatelet and anticoagulant use, American Society of Anaesthesiologists (ASA) grade, and body mass index (BMI). Operative data collected included operation start time, surgeon seniority, and drain insertion. Post-operative data analysed included post-operative complications, date and time of discharge, readmission within 30 days, and mortality. Pathological data collected included specimen weight and histology.

Data were collected and analysed by the authors using the trust electronic note systems in April 2019. This audit was registered with the Local Trust Audit Department, and ethical approval was not required.

## Results

A total of 140 cases were identified through coding in the period of January 2017 to December 2018, with 125 meeting inclusion criteria; 15 exclusions were made: three cases of incorrect coding, six cases of completion thyroidectomy, four cases of as part of another head and neck procedure, and two cases of emergency hemithyroidectomy.

Baseline characteristics are presented in Table 1. The mean age of this cohort was 51.9 years (range: 20-88 years), with 76% (n=95/125) female cases versus 24% (n=30/125) male cases. Indication for thyroidectomy included 23 (18.4%) compressive symptoms, four (3.2%) recurrent cystic swelling, and 98 (78.4%) due to ultrasound and/or fine needle aspiration characteristics which met criteria for thyroid lobectomy.

**Table 1:** Baseline characteristics ASA, American Society of Anaesthesiologists; BMI, body mass index

	Mean (range)
Age	51.9 (20-88)
BMI	26.4 (18-48)
ASA grade	1.9 (1-3)

Post-operative complications are listed in Table 2. Post-operative complications included five (n=5/125; 4%) cases of post-operative haematoma; one (n=1/125; 0.8%) case of pneumonia, four (n=4/125; 3.2%) cases of unilateral vocal cord palsy, and three (n=3/125; 2.4%) cases of wound infection. Four (80%) out of five post-operative haematoma developed within 24 hours of surgery and were returned to theatre during the same admission. One (20%) case represented to the accident and emergency department on day 6 post-operatively and was then taken to theatre for evacuation of haematoma. Patient characteristics of all post-operative haematoma cases are presented in Table 3. All cases that developed post-operative haematoma in our cohort were euthyroid, did not take anticoagulants or antiplatelets, and had no recorded retrosternal extension.

**Table 2:** Post-operative complications

	N (%)
Mortality	0 (0%)
Readmission < 30 days	1 (0.8%)
Haematoma	5 (4.0%)
Return to theatre	5 (4.0%
Wound infection	3 (2.4%)
Unilateral vocal cord palsy	4 (3.2%)
Pneumonia	1 (0.8%)

**Table 3:** Post-operative haematoma cases ASA, American Society of Anaesthesiologists; BMI, body mass index

	Case 1	Case 2	Case 3	Case 4
Age	75	52	45	70
Sex	F	F	M	F
Side of surgery	Right	Left	Left	Right
Reason for surgery	Compressive thyroid nodule	Thyroid nodule	Thyroid nodule	Thyroid nodule
Comorbidities	Lupus, hypertension, lymphoma	Alcohol excess, depression	Epilepsy, previous temporal lobectomy	Chronic obstructive pulmonary disease, lymphoma
Antiplatelet/anticoagulant use	Nil	Nil	Nil	Nil
ASA score	2	2	2	3
BMI	20	28	34	29
Primary surgeon seniority	Consultant	ST7	ST8	ST8
Intra-operative complication	Nil	Nil	Nil	Nil
Drain insertion intra-operatively	No	No	No	No
Thyroid weight	26.2g	5g	31g	6.4g
Post-operative sequelae	Cervical swelling noted 19 hours post-operatively during overnight admission; returned to theatre	Cervical swelling noted 10 hours post-operatively; returned to theatre	Cervical swelling on day 6 post-operatively; returned to theatre	Cervical swelling developed during inpatient stay; returned to theatre within 24 hours of initial operation

Mean in-patient stay for this cohort was 1.4 days, and mean hours in hospital from theatre start time to discharge were 34 hours. Two (n=2/125; 1.6%) cases were discharged on the same day within 9 hours post-operatively, with no reasons provided for their early discharge. No post-operative complications were recorded in these two cases. One (n=1/125; 0.8%) case was readmitted within 30 days, and this was one of the cases that had developed post-operative haematoma and required return to theatre.

## Discussion

This single-centre retrospective audit showed an overall complication rate of 15.2% (n=19/125). There were four (3.2%) recorded cases of post-operative haematoma that developed within the immediate post-operative period prior to discharge. All of these cases required return to theatre for evacuation of haematoma within 24 hours of initial surgery. These cases highlight the potential implications and risk associated with day-case surgery for thyroid lobectomy. One (0.8%) case developed a haematoma on day 6 post-operatively and was readmitted for surgery; this was the only case to develop a haematoma after 24 hours following initial surgery in our cohort.

A recent meta-analysis identified statistically significant risk factors for the development of post-operative haematoma in thyroid surgery as male gender, older age, Graves’ disease, hypertension, antithrombotic use, and low-volume hospitals [2]. Similarly, a large retrospective analysis of the UK national database for thyroid surgery identified male sex, age, and goitres as risk factors for post-operative haematoma [10]. Comparing these findings to our cohort, male gender was not an observable risk factor; the majority (n=4/5; 80%) of post-operative haematoma cases were observed in female patients. Only one (n=1/5; 20%) case complicated by post-operative haematoma had hypertension versus 29 (n=29/125; 23.2%) cases in this cohort. All cases that developed post-operative haematoma in our cohort were euthyroid and did not take anticoagulants, therefore excluding Graves’ disease and antithrombotic use, respectively, as risk factors in our cohort. Additionally, our centre is not a low-volume centre according to the definition in the meta-analysis [2], and therefore this risk factor was not relevant to our cohort. Finally, age as a potential risk factor for post-operative haematoma could be relevant to our cohort given that the mean age overall was 51.9 and was higher in the post-operative bleed cases (57.6).

The BAETS position statement on day-case thyroid lobectomy does include risk factors that increase the risk of haemorrhage [9], such as retrosternal goitre, perioperative anticoagulant or antiplatelet therapy, and re-operative same side surgery. None of the cases in our cohort that developed post-operative haematoma demonstrate these risk factors and therefore may have been "low-risk" when considered for day-case surgery. Additionally, BAETS recommend a minimum post-operative stay of 6 hours when considering day-case surgery in low-risk patients. All of the cases in our cohort that developed post-operative haematoma developed at least 6 hours or more after initial surgery, therefore emphasising the importance of a minimum stay being considered in day-case surgery.

A proposed risk factor from our cohort is ASA grade. All post-operative haematoma cases were ASA 2 or above which means they have a “moderate but definite systemic disturbance” identified from their pre-operative screening [11]. These patients are therefore already considered at an increased risk of developing post-operative complications [12]. In this audit, there were 27 (21.6%) cases who were ASA 1. None of these cases developed post-operative haematoma, one (3.7%) case developed right-sided vocal cord palsy, and one (3.7%) case developed partial left-sided vocal cord palsy. Given the low rate of complications and no patients developing post-operative haematoma in ASA 1 cases, it is possible that ASA 1 cases could be considered for day-case surgery. A larger sample size and statistical analyses could test this hypothesis.

Statistical analyses are beyond the scope of this audit, and it is therefore not possible to comment definitively whether cases of post-operative haematoma shared risk factors as identified in the aforementioned meta-analysis and review [2,10]. However, with the comparison of the frequency of these proposed risk factors across all cases versus post-operative haematoma cases as above, it is likely that age is the only predictable risk factor.

Limitations

This was a single-centre audit and therefore had a small sample size. This could be improved through collaboration with other centres.

## Conclusions

This audit concludes that given the unpredictable nature of post-operative haematoma, the potential risk to patients and medicolegal implications are too great to perform day-case thyroid lobectomy at our centre. It therefore remains best practice to follow current UK guidance and European consensus and continue to perform this procedure as an elective inpatient admission. Further research into the risk factors for developing significant post-operative complications would be beneficial and would help to identify low-risk patient populations who could safely undergo day-case thyroid lobectomy.
